# Stakeholders’ perspective on inclusion of key populations unique identifier codes in routine health information management system in South Africa

**DOI:** 10.4102/sajhivmed.v26i1.1727

**Published:** 2025-09-30

**Authors:** Mashudu Rampilo, Edith Phalane, Refilwe N. Phaswana-Mafuya

**Affiliations:** 1 Department of Environmental Health, Faculty of Health Sciences, South African Medical Research Council, University of Johannesburg (SAMRC/UJ), Pan African Centre for Epidemics Research (PACER) Extramural Unit, Johannesburg, South Africa

**Keywords:** key populations, HIV, unique identifier code, routine health information management system, South Africa, district health information system, health patient registration system, UNAIDS 95-95-95 targets, stigma and discrimination, data privacy

## Abstract

**Background:**

The global community has set an ambitious goal of ending HIV as a public health risk by 2030. To achieve this, South Africa must have a robust routine health information management information system (RHIMS) that provides programmatic data disaggregated by key populations (KPs) to enable effective HIV response.

**Objectives:**

To explore key stakeholders’ perspectives regarding the incorporation of KPs unique identifier codes (UICs) in the RHIMS in terms of opportunities, procedures, vulnerabilities, challenges, and considerations for enhancement in tracking the HIV care cascade in South Africa.

**Method:**

We conducted an exploratory, descriptive study that had three phases. First, we conducted stakeholder analysis and mapping using the power-interest matrix (Phase one). Second, we performed a qualitative document analysis (Phase two). Third, we conducted in-depth interviews with 20 stakeholders (Phase three).

**Results:**

We mapped 100 stakeholders according to their power and interest regarding the KPs UICs inclusion in RHIMS, with the South African National AIDS Council and the National Department of Health being the primary stakeholders. Stakeholders highlighted the KPs UIC facilitators as District Health Information System (DHIS) policy support, integration with TIER.Net and DHIS, data security, improved monitoring and evaluation, and KP-targeted programming. Stakeholders also cited resistance to change, stigma and discrimination, data privacy, and security as key concerns for the inclusion of KPs UICs in the RHIMS.

**Conclusion:**

Stakeholders support the inclusion of KPs UICs in public health data collection tools, emphasising its role in improving monitoring and evaluation, resource allocation, and KP-specific programming.

**What this study adds:** This study’s findings provide valuable insights into how the KPs UICs can be included in South Africa’s RHIMS, highlighting challenges such as stigma, resource constraints, and data privacy concerns. The study highlighted the importance of stakeholder collaboration and leveraging existing systems.

## Introduction

In 2015, the global community committed to eradicating AIDS as a public health threat by 2030.^1^ South Africa is among the countries actively working towards the attainment of the Joint United Nations Programme on HIV/AIDS (UNAIDS) 95-95-95 targets, which seek to ensure that 95% of individuals living with HIV are aware of their HIV status, 95% of those diagnosed are initiated on antiretroviral therapy (ART), and 95% of individuals on ART achieve sustained viral load suppression.^2^ In 2021, an estimated 70% of new HIV infections globally occurred among key populations (KPs) and their sexual partners, while in sub-Saharan Africa, they accounted for 51% of new cases, reflecting the disproportionate burden of HIV and the variation in prevalence between KPs and the general population across the region.^3,4^ The WHO defines KPs as men who have sex with men (MSM), sex workers (SW), people who inject drugs (PWID), and transgender (TG) people.^5^

In South Africa, about 7.8 million people are HIV positive, and 5.9 million are receiving ART, mainly through public health facilities.^6^ HIV prevalence differs significantly across KPs, with around 56% among SW, 26.9% among MSM, 51.9% among TG women, and 49.2% among PWID,^7,8,9^ while 75% of KPs access health services from public health facilities.^10^ Despite this disproportionate burden, the routine health information management system (RHIMS) in South Africa does not currently incorporate a unique identifier code (UIC) to systematically track HIV outcomes for KPs. This limitation hinders the ability to monitor service uptake and health outcomes effectively.

Integrating KPs UICs into RHIMS is key for targeted interventions, data accuracy, and inclusivity.^11,12^ The WHO guidelines outline a comprehensive package of services tailored to the needs of various KPs, such as condom and lubricant programming, HIV testing and counselling, HIV prevention and treatment, management of co-infections, and harm reduction for PWID. The WHO highlights the use of disaggregated data in achieving Sustainable Development Goals and improving KP health outcomes.^13,14^

Sub-Saharan African countries, including Burundi,^15^ Kenya,^16^ Malawi,^17^ and Liberia,^18^ have implemented UICs for KPs and demonstrated notable progress toward achieving the UNAIDS 95-95-95 targets. The use of UICs in these countries highlights the importance of KP-sensitive information systems and presents a strong rationale for South Africa to prioritise the integration of KPs UICs into its RHIMS as part of a more inclusive and effective national HIV response. The implementation of KPs UICs within South Africa’s RHIMS is largely constrained by a lack of political commitment to align the national HIV response with KP priorities and to allocate sufficient funding accordingly. The lack of disaggregated KP data in South Africa might hamper progress tracking toward the 2030 targets of ending AIDS.^19^ South Africa can benchmark best practices for KPs UICs inclusion into RHIMS by looking at countries such as Ghana and Kenya, which have successfully integrated UICs into their health systems.^20,21^

In South Africa, routine HIV data is initially recorded on paper-based clinical stationery by healthcare workers during the consultation or contact with the client, before being entered into the Three Integrated Electronic Registers (TIER.Net) by data capturers.^22^ The TIER.Net system serves as the electronic patient database within public health facilities, supporting the consolidation of HIV data at the facility level. Encrypted dispatch files containing patient-level information are exported from TIER.Net and uploaded to the web-based District Health Information System (DHIS2) at sub-district, district, and, where feasible, facility levels.^23^ The DHIS2 is utilised as RHIMS to collect, collate, analyse, and present routine health data.^24^ All public health facilities are mandated to report on indicators according to the National Indicator Data Set, which provides the indicator definitions.^25^ However, the District Health Information Management System (DHIMS) policy does not guide the flow of KP data from private and non-governmental organisations (NGOs) into the DHIS2 platform. This gap limits the integration of comprehensive HIV data needed for monitoring and responding to the needs of KPs. Recent findings on the South African health system assessment also recommended that monitoring and evaluation systems must be regularly reviewed.^26^ The National Department of Health (NDoH) has also introduced the Health Patient Registration System (HPRS) to uniquely identify individuals within the health system.^27^ The HPRS is a digital system that assigns a UIC to individuals accessing public health services. The HPRS is part of South Africa’s broader eHealth strategy aimed at improving continuity of care, reducing duplication of patient records, and enhancing data quality across the health system. While the HPRS enables patient tracking across facilities, it does not distinguish KPs from the general population, limiting its utility for disaggregated monitoring and evaluation of HIV services. It is crucial to understand the key stakeholders’ perspectives in addressing this gap.

Gathering stakeholders’ inputs requires proper stakeholder mapping and analysis to ensure maximum involvement of relevant stakeholders.^28^ The South African National AIDS Council (SANAC), which is the coordinating body for the country’s HIV response, plays a significant collaborative role by connecting government departments, civil society, and implementing partners. The SANAC also oversees the implementation of the ‘National Strategic Plan for HIV, TB, and STIs 2023–2028’.^29^

The KPs programmes in South Africa are implemented by the NDoH in collaboration with various implementing partners, including the Networking HIV & AIDS Community of South Africa, Beyond Zero, AIDS Foundation of South Africa, Wits Reproductive Health and HIV Institute, Anova Health Institute, TB/HIV Care, Aurum Institute, and Right to Care, among others. The majority of the KP programmes in South Africa are funded by international organisations such as the Global Fund, Centers for Disease Control and Prevention (CDC), and the United States Agency for International Development^30^ (USAID), and they make use of KPs UICs. Nonetheless, these donor-funded information systems are not integrated with the NDoH’s RHIMS.^31^ Strengthening the country’s public health monitoring and evaluation systems is increasingly critical, especially as public health facilities are now expected to absorb KP services previously supported by donor-funded programmes. This urgency follows an Executive Order by the United States of America Department of State to mandate the immediate suspension of all foreign aid activities funded through the President’s Emergency Plan for AIDS Relief (PEPFAR).^32^

To this end, we conducted a formative assessment to first identify and map stakeholders according to their influence and interest regarding the KPs’ UICs inclusion on RHIMS. Second, we analysed existing documents, tools, and guidelines to get insight into how different documents outlined KPs UICs integration. Third, we conducted in-depth interviews with stakeholders to understand their perspectives on the incorporation of KPs UICs in RHIMS, associated challenges, opportunities, and key considerations.

## Research methods and design

### Overview

**FIGURE 1:**
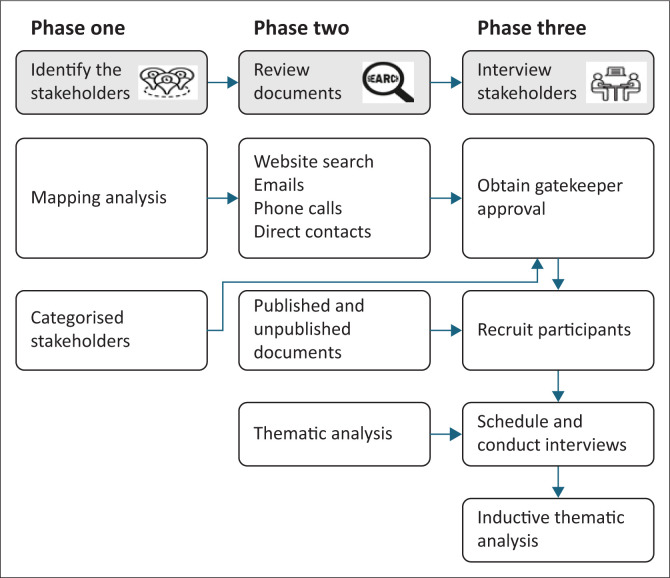
Multiphase study design for stakeholder engagement and data collection.

We have conducted a multiphase study including stakeholder mapping and analysis (Phase one), document review (Phase two), and stakeholder interviews (Phase three), as shown in Figure 1. The details are described elsewhere.^33^

### Phase one: Stakeholder mapping and analysis

#### Approach

Stakeholder mapping and analysis were conducted to compile a comprehensive list of relevant stakeholders and to summarise their roles concerning KP data within the RHIMS context. This process facilitated the strategic selection of interview participants representing a range of views, perspectives, and areas of expertise.

#### Study settings

The stakeholder mapping and analysis involved organisations based in Gauteng province, South Africa. Gauteng is the most populated province, with an estimated population of 15.9 million. The province covers approximately 18 178 km^2^, with three metropolitan municipalities (Ekurhuleni, Johannesburg, and Tshwane) and two districts (West Rand, and Sedibeng).^34^ The province was selected since most implementing partners reside in Gauteng. These implementing partners include the Networking HIV & AIDS Community of South Africa, Beyond Zero, AIDS Foundation of South Africa, Wits Reproductive Health and HIV Institute, Anova Health Institute, TB/HIV Care, Aurum Institute, and Right to Care, among others.

#### Participants

Stakeholders can be defined as actors who have an interest in the issue under consideration, and who are affected by the issue because of their position, including individuals, organisations, and networks.^30^ They were identified through a review of institutional roles, project documentation, and consultations with KP programme implementing partners, ensuring broad representation across government departments, civil society organisations, KP-led entities, healthcare providers, researchers, data managers, and funders. Many of these stakeholders collaborate with key institutions such as the SANAC, the NDoH, PEPFAR, the CDC, and the USAID. A power–interest matrix was used to analyse each stakeholder’s level of influence and interest in the integration of KPs UICs into RHIMS. Stakeholders implementing KP programmes have various data demands as they seek to measure the impact of HIV interventions for their programmes, and it is critical to map and analyse how best they can contribute to the RHIMS evaluation.^35^

#### Data collection

Stakeholder analysis and mapping were carried out between January and March 2024. An Excel spreadsheet was used to record stakeholders’ details (Online Appendix 1, Table 1-A1). These stakeholders operate within the national HIV architecture coordinated by the NDoH and the SANAC. As the principal coordinating body, SANAC convenes national platforms such as technical working groups, task teams, consultative workshops, and multi-stakeholder forums that guide the implementation of the national strategic plan (NSP) for HIV, tuberculosis (TB), and sexually transmitted infections (STIs) 2023–2028. During this phase, M.R. compiled a comprehensive list of stakeholders involved in KP programmes and RHIMS. Support for verifying each organisation’s relevance and contributing to the mapping of stakeholder roles within the broader landscape was provided by R.N.P.-M. and E.P. Following their identification, the stakeholders were mapped using a power–interest matrix to assess and categorise their level of power and interest in the implementation of KP data systems. The power–interest matrix, originally proposed by Mendelow,^36^ is a strategic tool used to assess and categorise stakeholders based on their level of power (influence over decisions) and interest (concern with outcomes). It supports the development of tailored engagement strategies by classifying stakeholders into four quadrants: manage closely (high power, high interest), keep satisfied (high power, low interest), keep informed (low power, high interest), and monitor (low power, low interest)This initial phase was essential for compiling a comprehensive list of stakeholders involved in KP programmes and RHIMS, ensuring the inclusive selection of participants for Phase three from the most relevant organisations.

### Phase two: Document review

#### Approach

A targeted document review was conducted to complement stakeholder insights and provide contextual understanding of RHIMS and KP data integration.

#### Inclusion and exclusion criteria

The review included English-language documents published between 2013 and 2023, sourced from South African government and NGO websites, as well as key multilateral organisations, including the Global Fund, UNAIDS, WHO, CDC Africa, and PEPFAR. Documents published before 2013 were excluded as the content might be outdated. Documents in languages other than English were also excluded.

#### Document search and extraction

Documents for review were sourced through online searches and direct requests from stakeholders. To access relevant grey literature, individuals within government and partner organisations were contacted to obtain unpublished reports. Data search was conducted between April and July 2024 through internet searches and direct correspondence with stakeholders. A structured Microsoft Excel tool was used to extract key information such as document title, source, publication year, purpose, target audience, and a summary of relevant content (Online Appendix 1, Table 2-A1).

#### Data analysis

For the document review, the Read materials, Extract data, Analyse data, and Distil (READ) approach was used as guidance to gain the most out of documents and ensured rigour in this document analysis.^37^

### Phase three: In-depth interviews with stakeholders

#### Study design and setting

A qualitative exploratory design was used. The qualitative research method was particularly appropriate for exploring complex, context-specific issues such as stakeholder experiences, perceived challenges, and opportunities related to the inclusion of UICs. Qualitative interviews allowed for the collection of rich, nuanced data that would not have been captured through structured quantitative tools. Moreover, the approach enabled the researchers to explore diverse viewpoints across multiple sectors, providing insight into policy, technical, and implementation dynamics that inform KPs’ HIV data governance and programmatic decision-making. The study was done in Gauteng province.

#### Sampling

A purposive sample of 20 key stakeholders was used to conduct the in-depth interviews. The sample was selected from the stakeholders who were mapped in Phase one.

#### Participants

In-depth interviews were conducted with stakeholders to explore their experiences and perspectives on the feasibility and implementation of KPs UICs within RHIMS.

#### Data collection

Face-to-face in-depth interviews were conducted using guiding questions with the 20 stakeholders from August 2024 to January 2025. The interview guide aided in examining the stakeholders’ familiarity with national data policies and tools, including the DHIMS policy. It also sought to assess the availability, accessibility, and use of KP-specific data, and to elicit views on the feasibility and implications of implementing a standard UIC for KPs. Furthermore, perceived barriers to UIC integration and recommendations on key requirements for effective implementation were explored. The interview guide was developed by consulting existing literature and engagements with key stakeholders by the first author (M.R.) under the supervision of the second (E.P.) and third (R.N.P.-M.) authors. The third author (R.N.P.-M.) has over 20 years of experience leading, executing, and implementing HIV and KP research. Before data collection, written permission was obtained from the respective organisations where participants were recruited. Participation was voluntary, and written informed consent was obtained before taking part in the study and for audio recording. Interviews were scheduled according to participants’ location preferences and conducted in private office spaces to ensure confidentiality. Each session involved only the participant, the interviewer, and a trained note-taker. Interview notes were taken to complement recordings in case of technical issues. Interviews were conducted in English and lasted between 20 and 40 min for each participant. The discussions focused on challenges, opportunities, and key considerations for integrating KPs UICs into the RHIMS.

#### Data analysis

The qualitative data were analysed using ATLAS.ti, version 22.^38^ The six stages of the thematic approach, comprising familiarisation with data, coding, identification, review, naming of major themes, and compiling the final report, were followed to maintain high study rigour.^39^ The recorded interviews were transcribed and analysed using inductive thematic analysis.^40,41^ All interview transcripts were independently coded by two researchers, followed by iterative discussions to refine and consolidate a shared coding framework. This collaborative process resulted in a final codebook. To assess the reliability of this coding framework, the researchers implemented a code–recode procedure. After a 1-week interval from the initial coding, two researchers independently recoded the same transcripts. Discrepancies in coding and interpretation were resolved through discussion until consensus was reached. Analytical rigour was further enhanced through the involvement of a third researcher, who reviewed the coded data and contributed to the development of the themes.

### Trustworthiness

The trustworthiness of this study was maintained throughout the research process and was confirmed through Lincoln and Guba’s five epistemological standards, including (1) credibility; (2) transferability; (3) dependability; (4) confirmability; and (5) authenticity.^42^ Credibility was established through triangulation across stakeholder mapping, document review, and in-depth interviews, as well as member checking during the analysis phase. Transferability was supported by providing rich descriptions of the study setting, participant roles, and context to allow readers to assess applicability to other settings. To ensure dependability, we maintained detailed records of the research process, applied a code–recode strategy, and involved multiple coders in developing and validating the coding framework. Confirmability was strengthened by using direct quotes to ground interpretations in participant voices and by keeping an audit trail of decisions made during data collection and analysis. Finally, authenticity was ensured by capturing a diverse range of stakeholder perspectives across sectors, roles, and KP affiliations, thus reflecting the complex realities of KPs’ data integration in RHIMS.^42,43^

### Ethical considerations

Ethical clearance to conduct this study was obtained from the University of Johannesburg Faculty of Health Sciences Research Ethics Committee (reference number: REC-2518-2023).

## Results

The results of this study are presented in three sections, as we have conducted stakeholder analysis and mapping, document analysis, and stakeholder interviews.

### Phase one: Stakeholder analysis and mapping

**FIGURE 2:**
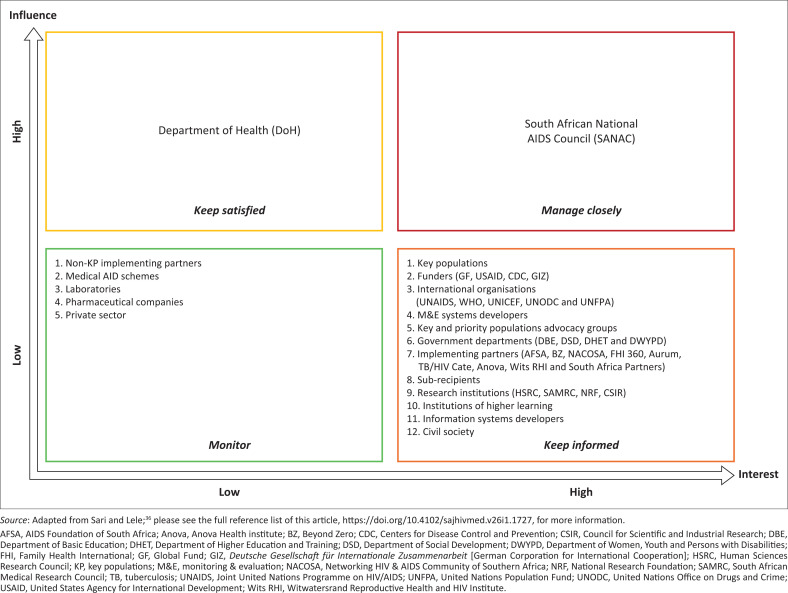
Stakeholder power–interest matrix.

We identified 100 stakeholders from different entities including national government entities such as the NDoH and Department of Social Development, Department of Basic Education, international organisations such as the WHO, UNAIDS, Global Fund and PEPFAR (USAID and CDC), as well as local partners engaged in KP advocacy, HIV programming and research. The power–interest matrix analysis shows that SANAC has the influence and interest to include KPs UICs on the national RHIMS (Figure 2). Although the NDoH is the custodian of health data, they are in a good position to influence change, but it lacks interest. Most of the stakeholders have a high interest and little influence.

### Phase two: Document review

The data obtained from the document review provided insight into how KP HIV data collection and reporting were recognised and operationalised across national and organisational documents. Eligible documents included policies, guidelines, protocols, reports, programme logs, performance ratings, funding proposals, meeting minutes, and strategic plans related to RHIMS and KP HIV programming, in both hard copy and electronic formats. The extracted fields included: document name, document type, source of the document, year of release, stated purpose, target audience, and a summary of key content related to KP HIV data reporting and integration within RHIMS (Online Appendix 1, Table 1-A1).

A total of 13 documents were assessed, including implementation guidelines (4), reports (6), an advert for a service provider (1), a student thesis (1), and a funding request (1). The documents were sourced from KP programme implementers (9), government department websites (2), funders (1), and university websites (1).

Two main themes and four sub-themes were identified through thematic analysis. The first theme focused on the implementation and functionality of unique identifiers, particularly the integration of KPs UICs within the HPRS (Table 1). Although the HPRS facilitates patient tracking across public health facilities, its inability to differentiate KPs from the general population limits its effectiveness for disaggregated monitoring of HIV services. The second theme highlighted the use of biometric systems as an alternative to alphanumeric UICs. Documents noted the advantages of biometric identification in enhancing data accuracy and safeguarding patient confidentiality. However, several technical challenges were reported, including system errors, low connectivity, and the absence of reliable backup mechanisms, all of which hindered effective implementation.

**TABLE 1:** Themes and sub-themes that emanated from thematic content analysis.

Themes	Sub-themes
1. Implementation and functionality of unique identifiers	1.1 Integration into National Health Information Systems
2. Use of biometric systems	2.1 Justification for biometric adoption 2.2 Data security and confidentiality 2.3 Technical and implementation challenges

#### Theme 1. Implementation and functionality of unique identifiers

The reviewed documents reflected a national commitment to advancing eHealth infrastructure by establishing a unique identifier for each patient. This initiative aligns with efforts to improve the country’s eHealth maturity and supports enhanced patient tracking across health facilities. The implementation of the HPRS was highlighted as a key mechanism to operationalise this goal, with the system intended to reduce patient loss to follow-up and strengthen continuity of care. However, a key limitation of the HPRS is its inability to distinguish KPs from the general population.

The country is aiming to move to the next eHealth ‘maturity level’ by establishing a unique identifier for each patient.^44^

Commitment to improving the use of unique identifiers through the implementation of the HPRS to track patients and prevent loss to follow-up.^45^

#### Theme 2. Use of biometric systems

Documents supported the utilisation of biometrics as opposed to alphanumeric UICs. This preference was largely driven by the need to ensure accurate tracking and avoid duplication in service delivery, particularly for mobile or marginalised populations who may access services at multiple sites.

Sex workers often provide different identification information to health service providers due to privacy concerns.^46^

Biometric systems were also seen as offering enhanced confidentiality and security compared to traditional identifiers. Unlike alphanumeric codes, biometrics reduce the risk of data manipulation or intentional misreporting, thereby strengthening the integrity of patient records and enabling more reliable longitudinal tracking.

Biometric databases are confidential and cannot be accessed by other partners or government agencies such as SAPS [*South African Police Service*] or South African Social Security Agency [*SASSA*].^47^

However, implementation challenges were noted from documents, particularly in large-scale grant-funded settings. Technical issues, limited connectivity, and inconsistent system performance frequently disrupted biometric registration and data reporting processes.

Biometric registration and reporting are a key element in the programme investments, but implementation was impacted by bugs, system rule errors, low connectivity, and inconsistent functionality.^48^

### Phase three: In-depth interviews

#### Participant characteristics

A total of 20 key stakeholders were interviewed in this study, and their demographic details are reflected in Table 2. Their roles were KP programme managers (*n* = 5), KPs (*n* = 6), monitoring and evaluation managers (*n* = 6), and software developers (*n* = 3). The KP programme managers were aged between 39 and 55 years, with 2 to 12 years of experience in KP programmes. They were employed by NGOs, parastatal organisations, and the government. The study also included KPs aged 24 to 39 years who were beneficiaries of KP programmes offered by NGOs. The KPs comprised female sex workers (FSW), MSM, people who use drugs (PWUD), and TG people.

**TABLE 2:** Demographic information for study participants.

Participant number	Sex	Age (years)	Current position	Years in this position	Type of employer
P01	Female	55	Deputy Director	13	Government
P02	Female	43	Technical Advisor	6	Non-government
P03	Female	39	Programme Specialist	6	Non-government
P04	Female	49	Programme Manager	2	Non-government
P05	Female	40	Programme Manager	11	Non-government
P06	Female	45	Assistant Manager	10	Government
P07	Female	45	M&E Specialist	9	Non-government
P08	Female	42	M&E Manager	2	Non-government
P09	Female	58	National M&E Officer	9	Non-government
P10	Male	42	M&E Manager	3	Non-government
P11	Male	35	M&E Coordinator	10	Non-government
P12	Male	34	KP	3	Non-government
P13	Male	39	KP	4	Non-government
P14	Female	33	KP	5	Non-government
P15	Female	39	KP	6	Non-government
P16	Bisexual	24	KP	1	Non-government
P17	Male	35	KP	4	Non-government
P18	Male	49	Software Developer	4	Non-government
P19	Male	55	Software Developer	9	Private
P20	Male	34	Software Developer	3	Private

M&E, monitoring and evaluation; KP, key population.

#### Results of inductive thematic analysis

Stakeholder interviews were conducted to explore in-depth perspectives, experiences, and insights related to the integration of KPs UICs within South Africa’s RHIMS. Given the complexity and sensitivity surrounding KP programming, stakeholder input was essential to understand contextual challenges, implementation barriers, and opportunities for system improvement. This approach allowed for the inclusion of diverse voices from policy, technical, and programmatic levels, ensuring that the analysis reflected both strategic and operational realities. Table 3 summarises the themes and sub-themes.

**TABLE 3:** Themes and sub-themes emanating from the inductive thematic analysis.

Themes	Sub-themes
1. Knowledge and perspectives on KPs data integration within existing policies	1.1 Familiarity with the district health management information system policy 1.2 Perceptions about DHIMS policy expansion 1.3 Familiarity with the NIDS review process 1.4 Perspectives about including KP indicators in NIDS 1.5 Perspectives on implementing one standard KPs UICs implementers 1.6 Perspectives on the inclusion of KPs UICs in public health data collection tools
2. Available KP data management tools	2.1 Awareness of data collection tools with KPs UICs 2.2 Accessibility of KPs’ HIV data 2.3 Use of KPs’ HIV programmatic data
3. Key considerations for KPs UICs inclusion in RHIMS	3.1 Resistance to change 3.2 Stigma and discrimination 3.3 Unauthorised access to KPs individuals’ data 3.4 Resources needed 3.5 Stakeholders’ involvement 3.6 Strong KPs UICs format 3.7 System design 3.8 Use of the biometric systems 3.9 Data privacy and security

KPs, key populations; DHIMS, district health information management system; NIDS, national indicator data set; RHIMS, routine health information management system; UIC, unique identifier code.

**Theme 1. Knowledge and perspectives on key populations data integration within existing policies:** This theme explored stakeholders’ awareness and understanding of key government data systems and policies that govern HIV data management in South Africa. It examined their familiarity with the DHIMS policy and the national indicator data set (NIDS), including the processes involved in indicator development and review. The theme also captured perceptions about expanding the scope of these systems to include data from private and NGOs, as well as views on the importance of disaggregating HIV data for KPs within national reporting frameworks.

Participants described varying degrees of knowledge of the DHIMS policy, with a range of views on whether these should be adapted to enable integrated collection of KP data through UICs.

Their familiarity with the DHIMS policy varied across different roles, with those from government structures being fully aware of DHIMS policy, while others had limited or no familiarity, as described below:

‘Yes, I have heard about the DHIMS policy. It is used to guide the collection and reporting of data from public health facilities.’ (P17, male, 35 years old)

‘Yes, I am familiar with DHIMS policy. The policy guides the data collection and reporting for health facilities, sub-district, district, up to national, with outlined activities, timelines, and responsible persons.’ (P10, male, 42 years old)

The scope of DHIMS policy does not cover KPs’ data flow from private and NGOs, and participants were asked for their views on including KP data from external stakeholders. Although most of the participants were not familiar with the DHIMS policy, some understood the need for its revision to include private and other NGOs who render services to KPs as well, noting that this would provide clear guidance to all groups collecting data and minimise the need for parallel reporting. Others, however, were opposed to the expansion of the DHIMS policy to cover private and NGOs, because of the DHIMS policy not applying to all areas:

‘Not sure if that is achievable because the DHIMS policy only covers [*the*] department of health facilities, and the data collection systems are standardized across the whole country. Implementing partners have different systems, which are donor-driven. Reporting mandates and indicator definitions for government, private, and NGOs are also not the same.’ (P08, female, 42 years old)

Stakeholders expressed different understandings of the NIDS review process, with some currently participating in NIDS revision meetings, and others previously exposed:

‘No, but I know it is the indicators that get reviewed by the Department of Health. I attended the meetings long ago, but now I am not involved anymore because of changing jobs.’ (P10, male, 42 years old)

While there was broad consensus on the need to include KP indicators in the NIDS, some concerns were raised by government officials regarding system capacity. Participants highlighted that the DHIS is already burdened with a high volume of indicators, which places strain on both data collectors and the system itself. As such, they suggested that reducing the number of existing indicators within NIDS should be prioritised before introducing additional disaggregation for KPs, to ensure sustainability and avoid further overwhelming the system:

‘No, the programme needs to cut on some of the indicators before we can make additions.’ (P06, female, 45 years old)

Stakeholders emphasised the importance of including KPs UICs in routine data collection tools to improve continuity of care and data accuracy. However, they also highlighted challenges, particularly the lack of standardisation across systems. The UIC formats currently used by the KP programme implementing partners are not aligned with those used in public health facilities. Stakeholders further noted that it is currently not possible to track clients referred to public health facilities using KPs UICs generated by implementing partners, resulting in a lack of continuity of care between KP programmes and government facilities. For example, SWs receiving services from KP programmes may be referred for ART or pre-exposure prophylaxis (PrEP), but once at the facility, they are recorded as part of the general population because of the absence of KP-specific identifiers on clinical stationery:

‘Yes, I do believe that we can have standard KP UIC. Yeah, if it’s standardised and it’s the same code everywhere the client goes, because if you think of the bank system, if you in Cape Town, you [*are*] still the same person, the information is the same, even if you go to Capitec bank while you are banking with FNB [*First National Bank*] it can work.’ (P08, female, 42 years old)

‘Yes, I think the country should consider that the implementing one standard Unique Identifier Code for key populations across all health data collection tools from government, private and NGO sector to avoid double counting of clients as key populations are highly mobile.’ (P17, male, 35 years)

**Theme 2: Available key population data management tools:** Stakeholders implementing KP programmes indicated that they have data collection tools that incorporate KPs UICs, including the risk assessment tool, which is used to evaluate clients’ risk of HIV acquisition and guide appropriate service provision. Other tools include the HIV testing form, referral form used to link clients to additional services or facilities, and screening tools for tuberculosis and STIs. The purpose of this question was to assess the structure and functionality of existing UIC formats, with the aim of informing potential adaptation and recommending a standardised format for national adoption:

‘The Global Fund [*GF*] tools like Reach, HIV testing services [*HTS*], TB [*tuberculosis*], STI [*sexually transmitted infection*], transfer, enrolment, service assessment, human rights, psychosocial services, and HIV risk assessment. These tools help us systematically document and track service provision for KPs while ensuring data consistency and confidentiality.’ (P07, female, 45 years old)

Stakeholders from the NDoH reported that data collection tools with tick boxes for KPs are currently being piloted in selected public health facilities referred to as KP Centres of Excellence. These clinics provide specialised, high-quality services tailored to the needs of KPs. While this initiative marks a positive step towards improved data collection, when data from these tools is aggregated for reporting purposes, information related to KPs is not disaggregated, limiting its effectiveness for targeted monitoring, planning, and service delivery:

‘Yes, we are piloting the data collection tool within KP centres of excellence, and we are currently assessing its effectiveness in capturing and managing UICs for improved service delivery.’ (P01, female, 55 years old)

Stakeholders implementing KP programmes reported that no document exclusively describes the steps for creating KPs UICs; however, the guidelines exist within the organisation’s data management tools. These internal guidelines vary across organisations and are often embedded within broader monitoring and evaluation protocols, which limit consistency in UIC application across programmes:

‘Not really, our tools are comprehensive, even if they don’t talk about how to create the UIC, it will be part of the main document, but not specifically talking about KP unique identifiers only.’ (P03, female, 39 years old)

The KPs’ HIV data accessibility varied due to it not being collected through government systems or only being available through private or non-profit partners who do collect it. As such, the use of KP HIV programmatic data varied among study participants depending on their occupation and where they were based:

‘Key populations’ HIV data is not easily accessible because government systems do not collect it. It is only available from partners implementing key population programmes.’ (P18, male, 49 years old)

‘Yes, for programme performance monitoring and reporting to the funder because they give us money to run the programme.’ (P04, female, 49 years old)

‘Yes, I do have access to KP data from the SANAC situation room, although it is not for the whole country. KP programmes are implemented in few districts, no data from public health facilities.’ (P17, male, 35 years)

On the other hand, the software developers indicated that they don’t use KPs HIV programmatic data:

‘No, we don’t use the data. We only manage the collection and reporting system. We don’t have permission to use it.’ (P20, male, 34 years old)

**Theme 3. Key considerations for key populations unique identifier codes inclusion on routine health information management system:** Stakeholders indicated that resistance to change among healthcare workers and government officials can undermine the successful adoption of new tools within the health system. They recommended conducting proper consultations and fostering stakeholder buy-in early in the process to reduce reluctance and promote ownership of revised data systems:

‘The challenge is that healthcare workers are afraid of change. They might be reluctant to adjust to the revised tools. If you can introduce something, certain people won’t like it, and no one can force them. Proper consultations with key stakeholders are important to avoid resistance.’ (P16, bisexual, 24 years old)

‘Challenges you can expect in the inclusion of Key Population Unique Identifier Code in Routine health information systems include a lack of buy-in from government officials, as they are the custodians of the health information system. You might encounter resistance to change.’ (P10, male, 42 years old)

One stakeholder representing PWUD cited a lack of trust in healthcare workers, as KPs mostly face judgment and discrimination at health facilities. Most of the PWUD clients stay in the streets and under the bridges without proper resources for bathing, and when they visit health facilities, some clinicians do not welcome them because of their unpleasant smell, which leads to them underutilising healthcare services because of the fear of stigma and discrimination:

‘PWUD clients don’t go to the clinics due to stigma and discrimination. The person will have six or seven abscesses before they go to the clinic. He will go there once he sees that now my leg or my arm is about to be taken off. Then that’s when they visit the health facility. They fear stigma and discrimination. Clinicians point to PWUD in front of all the clients, say hey, you must go and bathe before you come to the facility, they don’t even care; they can just put on the bandage and let you go.’ (P13, male, 39 years old)

‘Key populations might be scared to disclose their identity as key populations due to fear of stigma and discrimination. It is unlikely that key populations will allow healthcare workers to identify them as key populations on government systems.’ (P12, male, 34 years old)

Stakeholders raised concerns that data could be accessed by law enforcement or other entities, leading to possible unintended consequences. This fear of exposure may discourage KPs from engaging with health services or disclosing accurate information, ultimately undermining the purpose of adopting the KPs UICs:

‘There are risks of data breaches that can expose sensitive information, leading to potential harm to individuals identified as part of key populations. Healthcare providers and staff may need training to understand the importance of unique identifiers and how to use them appropriately without perpetuating stigma.’ (P01, female, 55 years old)

‘Security measures for the data system are very important, as some people can steal data and use it for the wrong reasons. The LGBTQI [*lesbian, gay, bisexual, transgender, queer/questioning, and intersex*] communities are being raped as punishment for changing their sexual orientation. For key populations like people who use drugs, the police can also use that information to arrest them.’ (P03, female, 39 years old)

Participants also raised concerns about budget constraints, emphasising that the successful integration of KPs UICs into the health information system would require substantial investment. This includes procurement of computers and data management infrastructure, development and maintenance of appropriate software, recruitment and training of personnel, and ongoing information technology (IT) support. Without dedicated financial and technical resources, there is a risk that implementation may be fragmented or unsustainable, particularly in under-resourced facilities.^49^ Stakeholders further noted that collecting detailed information and constructing the KPs UICs may be perceived as additional workload by healthcare workers:

‘The systems, computers, and databases all need to be in place, and consider personnel, I mean, they’ve already been complaining. It would almost be like adding more work to them. I don’t know if they would have the capacity to collect all that information.’ (P02, female, 43 years old)

Study participants highlighted the importance of meaningful consultation with key role players as a critical consideration for successful implementation. They stressed that engaging stakeholders across government departments, implementing partners, civil society organisations, and representatives of KP groups is essential to ensure that the system is inclusive, contextually appropriate, and responsive to the needs of end users. Inclusive consultation was seen as a means of building trust, addressing concerns early, and fostering collective ownership of the system:

‘Addressing these challenges requires a thoughtful, comprehensive approach that includes stakeholders from key populations, healthcare providers, data protection experts, and policymakers. The purpose of collecting unique identifiers must be clear. There needs to be a commitment to using this data to improve health outcomes for key populations, rather than for surveillance or punitive measures. There could be resistance or a lack of understanding regarding the need for specialised identifiers.’ (P01, female, 55 years old)

Software developers highlighted the need for a robust and flexible system architecture that can support offline data capturing, particularly in settings with poor internet connectivity. They noted that many facilities, especially in rural or resource-limited areas, face intermittent access to stable networks, which can delay data entry and compromise data quality. An offline-capable system would allow for real-time service documentation and later synchronisation once connectivity is restored, ensuring continuity in data flow and reducing the burden on healthcare workers:

‘Offline capability whereby you can still capture in an offline mode and then once you get access to the internet, you can then upload the data.’ (P20, male, 34 years old)

Additionally, stakeholders emphasised that the system should be adaptable to reflect the dynamic nature of individual identities within KPs. They noted that individuals may change the group with which they identify over time. For example, a person may transition between categories such as SW, TG woman, or MSM. Therefore, the system should be designed to accommodate such changes without losing continuity in the UIC or compromising the individual’s confidentiality. Flexibility in updating or reclassifying client profiles was seen as essential for ensuring accurate data, responsive services, and respectful engagement with KPs:

‘Key populations can change from one key population to another, for example, an MSM can decide to be straight. Some key populations don’t understand who they really are and can switch from different groups. The system should be designed in such a way that clients can change the key population group they belong to.’ (P18, male, 49 years old)

Study participants also emphasised the importance of training healthcare workers to handle sensitive information securely. They noted that effective training should include not only technical skills related to data protection and confidentiality protocols but also sensitisation to the specific needs and vulnerabilities of KPs. Building the capacity of healthcare workers to manage sensitive data responsibly was viewed as critical to fostering trust, reducing stigma, and ensuring the ethical use of information in both clinical and data management practices:

‘I think open sensitisation sessions are important, where Key populations, healthcare workers, and other community members are trained/sensitised about key populations. Healthcare workers should be sensitised in the presence of key populations. Once key populations know that healthcare workers are sensitised, they will come to the clinic without fear of being criminalised or stigmatized.’ (P12, male, 34 years old)

‘OK, first, I think considerations should include training of healthcare workers. The front-line healthcare workers must be sensitised on the key populations programme, so that they accept them and complete the data collection tools properly.’ (P12, male, 34 years old)

Consideration to utilise a biometric system was mentioned as a potential solution to ensure accurate identification of KPs. Participants suggested that biometric tools, such as fingerprint or iris scanners, could help prevent duplication of records and improve patient tracking across different service delivery points. However, concerns were also raised about data protection, the potential misuse of biometric information, and the need for strong governance frameworks to safeguard clients’ rights and confidentiality. The adoption of biometrics was seen as feasible only if accompanied by robust legal, ethical, and technical safeguards:

‘So, it’s better to use a fingerprint, yes, it’s better to use fingerprint, because other people may collect medication using someone’s name. Using fingerprints will ensure that the right person is collecting the medication.’ (P15, female, 39 years old)

Stakeholders noted that the country is currently aligning unique identifiers for the general population through the HPRS and suggested that incorporating KPs UICs within this system would not present significant challenges. However, they acknowledged that the incorporation of KPs UICs is not currently viewed as a national priority:

‘A [*UIC*] system for the general population is still being developed, but once completed, adding KP UIC should not be a hassle.’ (P06, female, 45 years old)

## Discussion

The study aimed to carry out a formative assessment of RHIMS stakeholders to support the integration of KPs UICs. We have explored operational challenges, vulnerabilities, policy considerations, and opportunities for integrating KPs UICs into South Africa’s RHIMS through stakeholder mapping and analysis, document review, and in-depth interviews. Stakeholder mapping identified key actors and the need for coordinated engagement. The three phases of this study highlighted the need for KPs UICs for improved continuity of HIV care, enhanced data disaggregation, targeted programming, better HIV cascade monitoring, and alignment with global and local data standards.

The document reviews highlighted gaps in guidelines, limitations of the HPRS, and considerations for biometric systems. In-depth interviews revealed stakeholder perspectives on policies, data tools, and practical considerations for KPs UICs inclusion in RHIMS. Stakeholder analysis provided a systematic approach to identifying the relative influence and interest of key actors in the integration of KPs UICs, as also reported by other studies examining health information system reforms and multi-stakeholder engagement.^50^ This facilitated the development of targeted engagement strategies tailored to stakeholders’ functional roles and strategic priorities.

We identified 100 stakeholders, which were mapped using a power–interest matrix, and the results showed that NDoH, while highly influential, has low interest in KPs UICs integration. Studies conducted elsewhere also highlight the significance of identifying the groups mostly affected by HIV through assessing their contribution to new HIV infections, transmission, and AIDS-related deaths.^51^

Successful implementation of digital health initiatives depends on strong collaboration among a wide range of stakeholders, including government officials, implementing partners, technology partners, and KP communities.^52^ The results underscore that SANAC possesses both the influence and interest necessary to champion the inclusion of KPs UICs within South Africa’s RHIMS. In other country contexts, national AIDS councils have played a pivotal role in coordinating stakeholders and facilitating the adoption of KPs UICs in close collaboration with ministries of health. Countries such as Ghana, Uganda, Burundi, Kenya, Mali, Burkina Faso, Togo, Liberia, and Malawi have incorporated KPs UIC on RHIMS through working closely with the national AIDS council and the national ministry of health.^20,21,47,48,50,52,53,54,55^ The results indicated that SANAC has both the interest and the power to be a key influential stakeholder in the incorporated KPs UICs on RHIMS, therefore, it is important to ensure NDoH is kept informed always about the KPs UICs implementation, as they have the influence.

The document review showed that there is limited documentation on KPs UICs integration into RHIMS in South Africa. Because of the lack of national ownership and standardised protocols, KP programme implementing partners utilise organisation-specific KPs UICs formats for their programmes.^19^ Donor-funded partners such as the AIDS Foundation of South Africa^47^ and TB/HIV Care^46^ have piloted biometric systems for UICs, particularly in SW programmes. However, documentation detailing how these UICs were constructed was not accessible. Documents accessed indicated a strong commitment from implementing partners to support the national HPRS through biometric systems.^44,45,56^ However, the documents also highlighted key implementation challenges, such as poor connectivity, technical glitches in devices, and the resources required for successful rollout. While South Africa has not yet adopted a national framework for KPs UICs, countries such as Ghana and Kenya have published accessible documentation outlining the formulation and implementation of alphanumeric UICs.^20,21^ These examples may serve as useful references when South Africa reaches the stage of formalising its national guidance.

Software developers saw KPs UICs as crucial for person-centred case surveillance and patient monitoring, concurring with other studies.^57^ Stakeholders emphasised that a standard national KPs UICs could improve patient tracking across facilities, ensuring continuity of care and reducing data duplicates. This statement corresponds with other findings, which indicated that a lack of UIC leads to inaccurate loss-to-follow-up estimates and retention as patients move between health facilities.^6^ In South Africa, UICs are assigned in individual facilities, enabling patient tracking within a single facility but not across multiple locations because the computers are not networked.^51^ Drawing on the National Digital Health Strategy (2019–2024), strengthening interoperability and networking between facilities can support seamless individual tracking.^52^

A number of challenges were described by stakeholders. Privacy concerns were prominent, with fears of data misuse, particularly by law enforcement, mirroring findings from South Africa, as well as China and the United States.^58,59^ Stigma and discrimination in healthcare facilities emerged as major barriers to disclosing the KPs’ status and getting the KPs UICs consistent with other studies.^53,60,61^ Resource constraints, including inadequate IT infrastructure, poor internet connectivity, and limited budgets, further complicated UIC implementation, aligning with findings from studies done in Zimbabwe and West Africa.^3,49,54^ Capacity building is crucial for equipping healthcare providers with the necessary skills to protect data and manage KPs UICs systems effectively. Training healthcare workers to reduce stigma and discrimination was another key factor for the successful integration of KPs UICs.^60,61,62,63,64^ Participants also indicated that healthcare providers are overburdened and may perceive the addition of KP status information on data collection tools as additional tasks, reinforcing the need for early consultation and engagement to build ownership and reduce resistance to change.

Despite these challenges, opportunities were documented for integrating KPs UIC. Both stakeholders and reviewed documents emphasised the potential of leveraging the existing HPRS platform to incorporate KPs UICs. While stakeholders acknowledged that the integration would be technically feasible given ongoing alignment of unique identifiers for the general population, they acknowledged that KPs UICs are not currently prioritised at the national level. Documents such as the PEPFAR Country Operational Plan (2022)^45^ also expressed commitment to strengthening unique identifier systems through HPRS to improve patient tracking and continuity of care, indicating a strategic entry point for future integration of KPs UICs.^44^ This convergence highlights the HPRS as a strategic entry point for future integration of KPs UICs, particularly if aligned with broader digital health priorities and adequately supported through policy, infrastructure, and stakeholder engagement. This is supported by a study done in South Africa, which found that leveraging existing health systems, such as the HPRS, could be a cost-effective strategy for enhancing data accuracy and security in HIV programmes.^65^ Many stakeholders viewed new electronic medical records as the best way to integrate KPs UICs.^51,52^ Involving KPs in the system design was also seen as an opportunity, like in other countries.^59^ Reports from the Pan American Health Organization highlighted that integrating KP biometric data into public health programmes has effectively improved KP monitoring in Latin America.^66^ Similarly, a study done in Ethiopia underscored the importance of data integration systems in enhancing health programme efficiency and achieving global health targets.^1^ South Africa can better track the HIV epidemic and progress toward global targets, ensuring no one is left behind in the fight against HIV.

### Strengths and limitations

The strengths of this study lie in its comprehensive multi-phase approach, which combined stakeholder mapping and analysis, document analysis, and stakeholder interviews. The study’s inclusivity of various stakeholders, such as government officials, NGOs, KPs, and software developers, ensured diverse perspectives. A general limitation of this type of study is that, being qualitative, the findings may not be easily generalisable to broader populations. Given that the study was conducted within a single province, the transferability of the findings to other provinces may be limited. To enhance transferability, detailed descriptions of the study setting, participant characteristics, and implementation processes were provided to enable readers to assess the relevance of the findings to other settings. Despite these limitations, the study, being the first of its kind to be conducted in South Africa, contributes valuable insights to the body of knowledge on the inclusion of KPs UICs, offering crucial information to support the achievement of the 95-95-95 UNAIDS goals by 2030.

## Conclusion

This formative assessment demonstrated that integrating KPs UICs into South Africa’s RHIMS is both necessary and feasible, though currently underprioritised. Through stakeholder mapping, document review, and in-depth interviews, the study highlighted systemic gaps, implementation challenges, and emerging opportunities. While privacy, stigma, and infrastructural limitations persist, the potential to leverage existing systems, such as the HPRS, offers a strategic pathway for future integration. Coordinated engagement, national ownership, and targeted investment in technical and human resources will be critical to ensuring effective implementation. Drawing on regional and global experiences, South Africa has an opportunity to advance inclusive, accurate, and person-centred HIV data systems that leave no one behind.
